# Fourteen Years of Experience of Liver Transplantation for Wilson’s Disease; a Report on 107 Cases from Shiraz, Iran

**DOI:** 10.1371/journal.pone.0167890

**Published:** 2016-12-08

**Authors:** Kamran B. Lankarani, Seyed Ali Malek-Hosseini, Saman Nikeghbalian, Mohsen Dehghani, Mohammad Pourhashemi, Kourosh Kazemi, Parisa Janghorban, Maryam Akbari, Sulmaz Ghahramani, Bijan Eghtesad, Maryam Moini, Abbas Rahmi Jaberi, Alireza Shamsaifar, Siavosh Gholami, Fatemeh Rahmanian, Bita Geramizadeh

**Affiliations:** 1 Health Policy research center, Shiraz university of Medical Sciences, Shiraz, Islamic Republic of Iran; 2 Shiraz Transplant Research Center, Shiraz University of Medical Sciences, Shiraz, Islamic Republic of Iran; 3 Pediatric Gastroenterology and Hepatology Department, Shiraz University of Medical Sciences, Shiraz, Islamic republic of Iran; 4 Transplant Center, Cleveland Clinic, Cleveland, Ohio, United States of America; 5 Gastroenterohepatology Research Center, Shiraz University of Medical sciences, Shiraz, Islamic Republic of Iran; 6 Shiraz neurology research center, Shiraz University of Medical sciences, Shiraz, Islamic Republic of Iran; Universidad de Navarra, SPAIN

## Abstract

**Background and Aim:**

Liver transplantation is a potential cure for liver damage from Wilson’s disease but the course of neuropsychiatric manifestations after transplantation remains undetermined.

**Material and methods:**

In this study, data on all patients who’d received a liver transplant for Wilson’s disease at the Shiraz Organ Transplantation Center between December 2000 and March 2014 were reviewed and compared to data on a control group who’d received a liver transplant over the same period but due to other causes.

**Results:**

Out of 2198 patients who’d received a liver transplant in the period; 107 patients were diagnosed with Wilson’s disease (21 with fulminant hepatic failure); age of patient ranged from 5 to 59 years; 56.07% of patients in this series had some type of neuropsychiatric manifestation before transplantation, of which 66.67% showed improvement after the procedure. 18 patients had aggravation of neuropsychiatric symptoms after transplantation. These neuropsychiatric symptoms were mostly for anxiety, tremor and depression but there were four cases of new onset dysarthria, rigidity and ataxia in various combinations. Survival rates of 1-month, 1-year, and 5-years for patients with Wilson’s disease were 88%, 86%, 82%, respectively, evaluations were not statistically different from that of the control group.

**Conclusions:**

Liver transplantation showed good long-term results in patients with Wilson’s disease, even in those presenting fulminant hepatic failure. Neuropsychiatric manifestations normally show improvement after transplantation but in some cases new onset of manifestations occurred after successful liver transplantation.

## Introduction

Wilson’s disease (WD) is an autosomal recessive disorder, first described by Wilson in 1912. It was more than a century ago that the disease was first described but diagnosis and treatment of this potentially lethal disorder still presents many challenges.

The gene ATP7B is responsible for WD. It encodes a trans-membrane protein ATPase that transports copper into the trans-Golgi compartment and incorporates it into the plasma protein ceruloplasmin that then facilitates copper excretion [1, 2]. Impairment in ATP7B function results in deposition of copper in many vital organs including the liver and brain. Copper deposition in these vital organs caused by WD has a devastating effect [3, 4].

Neuropsychiatric involvement (NI) in WD is common and may be present in more than two thirds of patients [5, 6]. Its manifestation can range from subtle psychological disorder to a disabling condition such as ataxia.

Hepatic involvement in WD tends to start at a younger age compared to NI and its manifestation may range from asymptomatic liver enzyme elevation to chronic hepatitis, cirrhosis and fulminant hepatic failure (FHF)[7].

Prevalence of WD is estimated in 1 in 30,000 of the general population [3], and this figure may be higher in regions with higher incidence of marriage between cousins.

WD is a fatal illness if it remains untreated. The most effective treatment is administration of chelating drugs such as D- penicillamine, tetrathiomolybdate, trientine and zinc salts which block intestinal absorption of copper [2].

Despite medical treatment, some patients progress to liver failure or develop neurologic disability and then the only treatment option for these patients is liver transplantation (LT) that could be a lifesaving procedure under such circumstances [4, 8, 9]. Indeed liver transplantation in WD is a cure for underlying liver disorder and was one of the earliest indications described for LT [10–13]. However LT may not reverse permanent damage to the central nervous system. In this regard there is debate on the course of neurologic manifestations after LT with reported improvement in most cases and deterioration in at least some cases [14].

The experience of LT in WD is not high in many centers and there are few published reports on long term follow up of these patients, especially on neurologic manifestations of the condition.

Favorable survival after LT in WD has been reported in most series but it is dependent on the number of transplants and duration of the follow up as well as the number of cases presented with FHF and age of patient at onset [15–18]. Reports on long-term follow up of LT treatment for this fairly common liver disease are scarce in western Asia [19–21].

Although LT for WD is a potentially lifesaving treatment, it is not free from complications [22]. There is also some controversy with regards to performing LT in the presence of progressive neurological deterioration [4, 9]. WD is the most common metabolic disorder to cause liver failure in the center, this study was designed to report long-term survival and follow up of patients that received liver transplant for WD and its complications compared to other conditions.

## Material and Methods

Medical records of all patients diagnosed with WD who had transplantation between December 2000 and March 2014, in the Shiraz Organ Transplantation Center, Iran, were reviewed.

Leipzig criteria were used for patient diagnosis (except molecular testing for ATP7B mutations (which was not available)). In this scoring system, score of 4 or more is diagnostic for Wilson disease [23]. Patients presented with at least one episode of encephalopathy within 12 weeks after the onset of liver disease with INR >1.5 were considered FHF (19). Others were assigned as having chronic Wilson’s disease (CWD).

For each case of CWD, one patient with the same gender and age who had received transplantation within 15 days of the index case with diagnosis of cirrhosis due to other causes was randomly selected from the database with the use of random numbers and these patients were formed the control group.

All patients received immunosuppressive therapy according to protocol of the Shiraz Organ Transplantation Center, which included high dose steroids for three days, early start of mycophenolate mofetil and tacrolimus and in most cases, maintenance with 2 latter mentioned drugs. Target level of Tacrolimus in those without renal dysfunction was 8–11 ng/ml for first 2 months after surgery and in follow up, if patient had no evidence of rejection or renal failure then the dose of tacrolimus was decreased gradually and at the end of first year with target level of 4–6 ng/ml. Beyond one year the level was determined by patient situation. The present study was approved and supervised by the Ethics Committee of Shiraz University of Medical Sciences, Shiraz, Iran. Patient information was anonymzed and de-identified prior to analysis.

Collected data included patient age, gender, weight, complications, neurologic manifestations, and MELD/PELD (models for end stage liver disease, Pediatric End-stage Liver Disease) scores. Laboratory values were recorded at baseline and at 15 days and at the latest available follow-up; these were assessed and values were compared between groups in the study.

In this study, the main endpoint was survival of every patient, which was counted as a function of time after transplantation until the date of death or the end of the study (March, 2014). Data on deaths unrelated to liver disease or transplant procedure, and data on those patients still alive at the end of the study, were censored for cumulative survival estimation.

All statistical analyses were performed with the SPSS 20 statistical package. The continuous variables were summarized using Median [Inter Quartile Range IQR] and categorical variables were summarized using number (percentage). Mann-Whitney U test were used to compare outcomes on continuous measures and categorical variables were compared by Chi-square analysis. The Kaplan-Meier method was used to estimate probability of patient survival. In the Kaplan-Meier method, survival was computed according to different variables in a univariate manner and the Breslow test was used to make comparisons between the different categories of variables. P-values were two-sided using an alpha of 0.05 as the standard for determining significance.

## Results

During this period, out of 2198 patients who’d received LT at the center, 107 with WD diagnoses received liver transplant. 21 of these patients presented fulminant hepatic failure (FHF).

For each of the 86 patients with WD who presented chronic liver disease, one age-gender matched patient was randomly selected from those who were transplanted within 15 days before or after the index case. These 86 patients formed the control group for CWD. None of these patients were transplanted solely for neuropsychiatric manifestations.

Characteristics of the studied patients are shown in Table 1, S1 File. The median[IQR] of age was 16[9–21.5] years for patients presenting FHF and 22[15–26] years for those with CWD with a range of 5 to 59 years for all cases with WD (P-value = 0.006).

**Table 1:** Comparison of characteristics and complications in acute** and chronic WD patients with controls.

Characteristics	WILSON		CONTROL	P-value^1^	P-value^2^
	Acute	Chronic			
**Number**	21	86	86	-------	
**Age (year)**	16[9–21.5]	22[15–26]	21[14–26]	0.006	0.676
**Sex (M/F)**	13/8	51/35	51/35	0.08	1.000
**AST**	145[89–240]	77.5[50.7–132.7]	59[42.2–118.7]	0.004	0.063
**ALT**	91[47–237]	63.5[32–135]	66.5[37–122.5]	0.227	0.959
**AST/ ALT**	1.4[1.1–1.7]	1.3[0.95–1.9]	1.2[0.93–1.6]	0.632	0.197
**Alb**	3.1[3–3.5]	3.1[2.8–3.5]	3.2[3–4]	0.528	0.077
**INR**	5.7[2.7–6.5]	1.9[1.6–3.07]	2[1.5–3.3]	0.001	0.486
**ALK**	376[225–849]	429[249–670]	409.5[248.5–719]	0.865	0.808
**Creatinine, mg/dL**	0.2[0.1–0.5]	0.7[0.5–1]	0.6[0.3–1]	0.0001	0.225
**Total Bilirubin, mg/dL**	7[4.3–10]	4.9[2.4–9.6]	3[1.2–8]	0.132	0.040
**ALK/Total Bilirubin**	47.97[26.33–115.23]	87.98[42.2–197.05]	97.07[34.3–226.25]	0.146	0.621
**Weight before LT (kg)**	55[26–67]	62[51–75]	58.5[41.7–67]	0.111	0.045
**TSH**	4.7[3.04–5]	2.5[1.5–3.1]	1.8[1.2–2.6]	0.047	0.047
**WBC**	5800[1555.8–7250]	3700[2000–5800]	4850[3475–8975]	0.393	0.001
**PT**	33.5[19.9–37.7]	17[14.4–20.8]	17[14.7–19.9]	0.0001	0.761
**PTT**	43.5[30.2–63]	44.5[35.7–58.5]	42[36–52]	0.969	0.631
**PLT**	68000[114.6–96500]	61500[28250–100750]	83000[41500–159000]	0.913	0.006
**MELD score**	20.5[12.5–40]	20[17.7–24]	19.5[17–21.2]	0.727	0.484
**PELD score**	33[27–35.2]	24[18–29.5]	24[19.5–32.5]	0.188	0.968
**Hospital stay**	13[10.5–16.5]	13[11–19.2]	12[10–17]	0.611	0.137
Death*	4(19)	11(12.8)	18(20.9)	0.488	0.585

-Data expressed as Median [IQR], Number or Number (Percent).

* Death during the study (follow up duration: 4015 days).

** Acute liver failure due to WD.

-P-values calculated by Mann-Whitney, and Chi-square test.

-P-value^1^ Difference between acute and chronic Wilson’s disease

-P-value^2^ Difference between chronic Wilson’s and control groups

The main cause of cirrhosis in the controls was autoimmune hepatitis. Age, gender combination, hospital stay and the level of albumin, Alkaline phosphatatse(ALK), AST, ALT, Creatinine, AST/ALT ratio, MELD and PELD (when appropriate) were determined as not significant between case and control groups (P-values > 0.05). Total bilirubin was slightly higher in WD patients compared to those in the control group (4.9[2.4–9.6] vs 3[1.2–8], P-value = 0.040). Mean weight before transplantation and the level of TSH in cases were significantly lower in patients in the control group compared to those with CWD (P-value < 0.05).

Early and late complications in WD and the control group are shown in Tables 2 and 3.

**Table 2:** Comparison of early complications* in acute** and chronic WD patients with controls.

Complications	WILSON		CONTROL	P-value^1^	P-value^2^
	Acute	Chronic			
Acute rejection	3(14.3)	29(33.7)	13(15.1)	0.111	0.005
Renal failure	0(0)	8(9.3)	8(9.3)	0.351	1.000
Hypertension	8(31.8)	37(43)	43(50)	0.682	0.359
Proteinuria	4(19)	21(24.4)	29(33.7)	0.776	0.179
Hyponatremia	5(23.8)	20(23.2)	25(29.1)	0.957	0.386
Hyperkalemia	3(14.8)	10(11.6)	16(18.60	0.716	0.202
Leukopenia	2(9.5)	28(32.6)	29(33.7)	0.055	0.871
Thrombocytopenia	3(14.3)	24(27.9)	23(26.7)	0.267	0.864
Viral infection	0(0)	1(1.2)	0	1.000	1.000
Fungal infection	1(4.8)	2(2.3)	0(0)	0.484	0.497
Bacterial Infection	4(19)	7(8.1)	12(14)	0.221	0.224
Bowel perforation	0(0)	1(1.2)	1(1.2)	1.000	1.000
Retransplant	0(0)	1(1.2)	0(0)	1.000	1.000
Hepatic Artery thrombosis	1(4.8)	1(1.2)	0(0)	0.355	1.000

*Early complications are happened during one month after transplantation.

** Acute liver failure due to WD.

- Data expressed as Number (Percent).

- P-values calculated by Chi-square and Fisher exact test.

-P-value^1^ Difference between acute and chronic WD

-P-value^2^ Difference between chronic Wilson’s and control groups

**Table 3:** Comparison of late complications* in acute** and chronic WD patients with controls.

Complications	WILSON		CONTROL	P-value^1^	P-value^2^
	Acute	Chronic			
Rejection					
Acute rejection	6(35.3)	18(24)	16(23.5)	0.338	0.947
Chronic rejection	0	3(4)	4(5.9)	Ns	0.709
Renal failure	0	4(5.3)	3(4.4)	Ns	Ns
Biliary complication	0	7(9.3)	7(10.3)	0.341	0.847
Hepatic Artery thrombosis	0	0(0)	1(1.5)	Ns	0.476
Retransplantation	0	1(1.3)	1(1.5)	Ns	Ns
Fungal infection	0	1(1.3)	0	Ns	Ns
Bacterial infection	2(9.5)	6(8)	3(4.4)	0.637	0.499

*Late complications are happened after one month from transplantation.

** Acute liver failure due to WD.

- Data expressed as Number (Percent).

- P-values calculated by Chi-square and Fisher exact test.

-P-value^1^ Difference between acute and chronic WD

-P-value^2^ Difference between chronic WD and control groups

60 (56.07%) of WD patients in this series had some type of neuropsychiatric manifestation before LT. Of these patients after LT, neurologic manifestations improved in 40 (66.67%), 2 (3.33%) had no change and 18 (30%) of patients with pre LT NI had some type of exacerbation after transplantation, there were some cases of new onset of neuropsychiatric symptoms after LT in these series, which were mostly anxiety, tremor and depression (Table 4). There were also four cases of new onset dysarthria, rigidity and ataxia in variable combinations.

**Table 4:** Comparison of neurologic manifestations in Wilson patients before and after liver transplantation.

Neurologic complication	Pre-OLT	Post-OLT	P-value
Tremor	30(40.5)	13(17.6)	0.001
Incoordination	1(1.4)	1(1.4)	Ns
Dystonia	1(1.4)	0(100)	-
mask face	1(1.4)	1(1.4)	Ns
fine.m.task	13(17.6)	2(2.7)	0.001
Drooling	16(21.6)	4(5.4)	<0.0001
Dysarthria	16(21.6)	8(10.8)	0.05
Rigidity	11 (14.9)	1 (1.4)	0.006
Gait Disorder	13(17.6)	5(6.8)	0.008
Problem in School performance	1(1.4)	0(100)	-
Apraxia	2(2.7)	1(1.4)	Ns
Seizure	12(16.2)	0(100)	-
Ataxia	24(32.4)	8(10.8)	0.002
Drooling			
Anxiety	12(16.4)	10(13.7)	0.791
Depression	18(24.3)	4(5.4)	0.004
Compulsive	3(4.1)	1(1.4)	Ns
Phobia	4(5.4)	0(100)	-
Aggression	3(4.1)	2(2.7)	Ns
poor Memory	7(9.5)	1(1.4)	0.031
Neurosis	12(16.2)	10(13.5)	0.688
Psychosis	6(8.1)	4(5.4)	0.727

-Data expressed as Number (Percent). *P*-values calculated by McNemar's test.

Among the 107 patients with WD, 16 died during the study period. One death was caused by car accident, therefore omitted from the survival analysis. In the control group of 86 patients, 18 patients died during follow up. There was no statistical significant diffrence between patients that remained alive and those with WD that deceased in terms of frequency of outcome and post LT complications (P-values > 0.05) (except for acute rejection) (Table 5). Mean of FBS (Fasting Blood Sugar) in deceased patients in the group with WD were significantly higher than live patients. There were also more episodes of acute rejection among deceased patients. One case of bowel perforation occurred that resulted in death in the group of patients with WD.

**Table 5:** Demographic, clinical, and laboratory characteristics and complications of post transplantation in 107 Wilson patients who underwent liver transplantation by mortality.

Characteristics	Live patients (n = 92)	deceased patients (n = 15)	P-value
Age (year)	19[13–25.7]	22[17–28]	0.306
Male/Female	60/32	7/8	0.173
Hospital length of stay	13[11–17]	14[11–24]	0.669
MELD score	20[18–25]	21.5[19.2–25.5]	0.329
FBS	86[73–96]	145[86.2–218.7]	0.001
AST	93[53–151.5]	67[54–175]	0.630
ALT	77.5[35.2–138.7]	63[37–234]	0.953
AST/ALT	1.4[0.99–2]	1.2[0.95–1.4]	0.258
INR	2.1[1.6–4.9]	2.2[1.7–5.9]	0.585
BUN	9[6–14]	14.5[7.1–20.2]	0.066
ALK	453[249–762]	293[227–537.5]	0.191
Creatinine, mg/dL	0.7[0.4–0.9]	0.5[0.25–0.95]	0.472
Total Bilirubin, mg/dL	5.7[2.7–9.7]	5.4[1.7–9]	0.545
Acute rejection	22(23.9)	10(66.7)	0.001
Renal failure treated with dialysis	5(5.4)	3(20)	0.082
Hypertension	32(34.8)	5(33.3)	0.913
Proteinuria	22(23.9)	3(20)	Ns
Hyponatremia	20(21.7)	5(33.3)	0.325
Hyperkalemia	11(12)	2(13.3)	Ns
Leukopenia	25(27.2)	5(33.3)	0.622
Thrombocytopenia	22(23.9)	5(33.3)	0.436
Viral infection	1(1.1)	0	Ns
Fungal infection	2(2.2)	1(6.7)	0.367
Bowel perforation	0	1(6.7)	0.140
Retransplant	1(1.1)	0	Ns
Hepatic Artery thrombosis	1(1.1)	1(6.7)	0.262

**- Data expressed as** Median [IQR], **Number or Number (Percent)**.

*- P*-values calculated by Mann-Whitney, and Chi-square and Fisher exact test.

**Fig 1:**
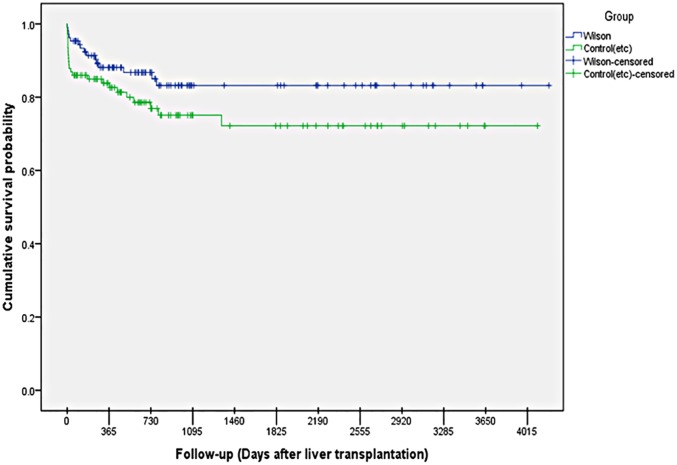
Kaplan-Meier estimates of patients’ survival after liver transplantation, stratified by cause of cirrhosis (Wilson’s disease vs. general liver transplantation). Wilson’s disease patients (blue line) and general liver transplantation population (green line).

The follow up duration was 4015 days. Overall survival rates until 20, March 2014, were 86%, and 77.6%, in WD and control groups respectively. The 1-month, 1-year, and 5-year patient survival rates for WD were 88%, 86%, 82%, respectively. Fig 1 displays Kaplan–Meier survival graphs for the WD patients and other liver transplant patients. In WD patients, mean survival was of 3544.5 days (95%CI of 3232.9–3856). In the control group, mean survival was of 3059.2 days (95%CI = 2685.3–3433.1). After submitting the above-mentioned results to a Breslow (Generalized Wilcoxon) test, there was no statistical significance between the groups (P-value = 0.098).

Based on the Kaplan-Meier method, there was no significant difference for survival between patients with acute liver failure due to WD and those with chronic WD (P-value = 0.449). The mean survival rates were 2891.5±220.2 and 3738.2±179.3 days for patients with acute liver failure due to WD and chronic WD respectively.

There was no statistical significant difference determined between donor characteristics and final outcome of patients with Wilson’s disease including: donor type (deceased vs live) (P-value = 0.20), gender (matched gender vs non matched) (P-value = 0.94), and age difference of donor and recipient(<20 &>20 years) (p.value = 0.40).

## Discussion

Liver transplantation is a cure for liver disease in cases of WD. There is a variety of hepatic presentation in cases of WD so diagnosis classes many patients on in the differential diagnosis list. The practice of consanguineous marriage through gene concentration may increase the probability of WD. There is currently a cultural transition taking place in Iran but the tradition of inter familial marriage is still common practice. This practice has contributed to a high rate of WD in Iranian society. Indeed at the Shiraz transplant center as the major referral center in the nation for LT, WD was the most common metabolic disease that led to the need for transplantation [20, 24].

Diagnosis of WD before transplantation was a major challenge, particularly in patients presenting FHF as the diagnostic tests including serum ceruloplasmin, urine and serum copper may be inconclusive. There were 21 cases of WD presenting FHF reported but this may have been an underestimation. Considering that there is a nearly 100% risk of death in WD presenting with FHF in the absence of LT, the condition should always arouse suspicion, especially in younger patients with FHF. The Median [IQR]age of patients with WD with FHF in the series was16[9–21.5]years compared to 22[15–26]years in patients presenting chronic liver disease (P-value = 0.006) highlighting the younger age of patients with FHF. There was a wide age range of patients with WD, with the youngest aged 5 and the eldest age 59 years. This indicates that WD should be classified in terms of differential diagnosis of liver disease even in those older than 50 years, particularly in the study region.

Median [IQR] of White blood cell count before LT were lower in patients with WD in chronic presentation compared to those of the control group (Table 1). This may indicate the effect of chelating agents before LT in CWD.

The hospital course and complications in WD presenting with FHF versus those with CWD as well as comparison with control group were comparable for most events (Tables 1–3). ALT level in patients with FHF was lower than what reported by other investigators. This might be related to later presentation of patients to our center. There was a higher rate of acute rejection in CWD compared to the control group [29(33.7%) vs 13(15.1%) P-value = 0.005]. As all patients in this group were receiving copper chelating agents before transplantation mainly D-pecillamineand for variable times, the immune modulating effect of this drug might have contributed to this higher rate of rejection [25]. The higher weight of patients with CWD compared to the control group may also have contributed to under dosage of immunosuppressive drugs or other mechanisms [26].

None of the patients in this study received transplantation only for NI; all had concomitant liver disease. Indeed 43.93% of WD patients had no neuropsychiatric manifestations before transplantation according to evaluation by a neurologist. The course of neurologic manifestations after LT was favorable with apparent improvement in 66.67%. Eighteen patients developed aggravation of neuropsychiatric manifestations after LT. Most of these were probably related to drugs (tremor) or a psychological reaction to major surgery (anxiety) or chronic care (depression). This group had no characteristics that distinguished them from others in statistical analysis. Prevalence of neurologic symptoms before LT in our series was comparable to that in other series that reported 60% prevalence, even in prospective studies [3]. This might be related to more intensive evaluation at our center, especially considering psychiatric manifestations. Our report on details of the evolution of neuropsychiatric manifestation after LT showed that much of the previous reported deterioration was unrelated to the natural history of LT in WD itself but was rather related to the side effects of drugs and the effect of major stress. [27,28]. As demonstrated, there was the possibility of new onset neurologic symptoms after LT but the risk was small [14, 22, 24–26].

Fifteen of the patients with WD in this study died after liver transplantation. Compared to those who were alive at the end of follow up, the deceased patients had higher FBS and higher rates of acute rejection but other adverse events showed no difference between alive and deceased group. Higher FBS in deceased WD cases could be attributed to higher doses of immunosuppressive drugs in this group compared to doses taken by survivors.

LT had a trend for better survival in patients with WD compared to the control group although the difference did not reach statistical significance. This was despite inclusion of 21 patients with FHF in the WD group. This trend for better outcome has been reported from some but not all other centers. The difference in survival rate reported from different centers was probably related to the number of patients presenting FHF, age of recipient and volume of transplant procedure at each center [15–18, 29].

Most death events in the series occurred in the 1^st^ year (early), after LT had been reported in previous studies [8].

This study had several limitations including its retrospective design and limitation in terms of confirming WD among patients presenting FHF that probably resulted in underestimation of WD in this group and lack of appropriate control for FHF group. Additionally we did not investigate the neurologic manifestations in our control group in a systematic manner. So we could not compare the post liver transplantation neurologic manifestations between two groups. However this experience for LT in 107 patients with WD in a single center in west Asia may help other centers with more limited experience in LT for WD.

In conclusion this study revealed a good survival after LT in WD with improvement in neuropsychiatric manifestations in most patients with WD.

## Supporting Information

S1 File Patients' characteristics. (XLSX)
